# Revealing hidden species diversity in closely related species using nuclear SNPs, SSRs and DNA sequences – a case study in the tree genus *Milicia*

**DOI:** 10.1186/s12862-016-0831-9

**Published:** 2016-12-01

**Authors:** Kasso Daïnou, Céline Blanc-Jolivet, Bernd Degen, Priscilla Kimani, Dyana Ndiade-Bourobou, Armel S. L. Donkpegan, Félicien Tosso, Esra Kaymak, Nils Bourland, Jean-Louis Doucet, Olivier J. Hardy

**Affiliations:** 1 Nature + asbl / TERRA Research Centre, Central African Forests, Gembloux Agro-Bio Tech, University of Liege, Passage des Déportés 2, 5030 Gembloux, Belgium; 2 Université d’Agriculture de Kétou, BP 43, Kétou, Benin; 3 Thünen Institute of Forest Genetics, Sieker Landstrasse 2, 22927 Grosshansdorf, Germany; 4 Kenya Forestry Research Institute, Biotechnology Section, P. O. Box 20412-00200 Nairobi, Kenya; 5 IRAF/CENAREST, BP 842 Gros-bouquet, Libreville, Gabon; 6 TERRA Research Centre, Central African Forests, Gembloux Agro-Bio Tech, University of Liege, Passage des Déportés 2, 5030 Gembloux, Belgium; 7 Evolutionary Biology and Ecology – CP 160⁄12, Faculté des Sciences, Université Libre de Bruxelles, Av. F. Roosevelt 50, 1050 Brussels, Belgium; 8 Service of Wood Biology, Royal Museum for Central Africa, Tervuren, Belgium

**Keywords:** Tropical forests, *Milicia*, Cryptic species, Species delimitation, SNP, Microsatellites, DNA sequences

## Abstract

**Background:**

Species delimitation in closely related plant taxa can be challenging because (i) reproductive barriers are not always congruent with morphological differentiation, (ii) use of plastid sequences might lead to misinterpretation, (iii) rare species might not be sampled. We revisited molecular-based species delimitation in the African genus *Milicia*, currently divided into *M. regia* (West Africa) and *M. excelsa* (from West to East Africa). We used 435 samples collected in West, Central and East Africa. We genotyped SNP and SSR loci to identify genetic clusters, and sequenced two plastid regions (*psbA-trnH*, *trnC-ycf6*) and a nuclear gene (*At103*) to confirm species’ divergence and compare species delimitation methods. We also examined whether ecological niche differentiation was congruent with sampled genetic structure.

**Results:**

West African *M. regia*, West African and East African *M. excelsa* samples constituted three well distinct genetic clusters according to SNPs and SSRs. In Central Africa, two genetic clusters were consistently inferred by both types of markers, while a few scattered samples, sympatric with the preceding clusters but exhibiting leaf traits of *M. regia*, were grouped with the West African *M. regia* cluster based on SNPs or formed a distinct cluster based on SSRs. SSR results were confirmed by sequence data from the nuclear region *At103* which revealed three distinct ‘Fields For Recombination’ corresponding to (i) West African *M. regia*, (ii) Central African samples with leaf traits of *M. regia*, and (iii) all *M. excelsa* samples. None of the plastid sequences provide indication of distinct clades of the three species-like units. Niche modelling techniques yielded a significant correlation between niche overlap and genetic distance.

**Conclusions:**

Our genetic data suggest that three species of *Milicia* could be recognized. It is surprising that the occurrence of two species in Central Africa was not reported for this well-known timber tree. Globally, our work highlights the importance of collecting samples in a systematic way and the need for combining different nuclear markers when dealing with species complexes. Recognizing cryptic species is particularly crucial for economically exploited species because some hidden taxa might actually be endangered as they are merged with more abundant species.

**Electronic supplementary material:**

The online version of this article (doi:10.1186/s12862-016-0831-9) contains supplementary material, which is available to authorized users.

## Background

Species diversification and morphological evolution are not always correlated, as demonstrated by the existence of hidden genetic diversity in taxa previously considered as single species. In sexual organisms, a cryptic species complex in the sense of [1] designates reproductively isolated species assigned to the same taxonomical species name because they are hardly distinguishable morphologically: sibling taxa with obscure morphologies [2, 3]. The concept is not new: in 1942, Ernest Mayr listed several known ‘sibling species’ in support to his criticisms of the morphological species concept. But the prevalence of hidden diversity in various taxa has been much better appreciated in the two last decades owing to surveys of DNA variation within species or closely related species [2]. The abundance of cryptic species raises several issues. Estimating the number of species has become a challenge [4, 5]. The use of confounding cryptic species as biological indicators or for medicinal applications can be detrimental if the cryptic species in question differ from their common allied species members in terms of their ecology or physiology. Conservation issues need to be considered for economically important species, such as timber tree species which are sometimes composed of complexes of cryptic species [2, 5].

Hidden genetic diversity can sometimes be detected through direct observations, suggesting for instance reproductive barrier between individuals (phenology or pollination patterns, etc.). Reproductive isolation between sympatric sibling species can be detected using genetic markers. In addition, several molecular markers are needed because different evolutionary processes such as incomplete lineage sorting can obscure the genetic divergence displayed by a single marker. DNA-based phylogenetic approaches are often used to delineate species, and reproductively isolated groups can be identified following the phylogenetic species concept if they segregate into different monophyletic lineages. However, the absence of monophyly is not conclusive because genetically isolated groups can be paraphyletic or polyphyletic [6, 7]. Hence, in order to delineate closely related species, methods that do not require monophyly would be suitable.

A variety of such methods have been proposed [3]. Flot et al. [8] suggested the construction of haplowebs of nuclear sequences to identify fields for recombination (FFR, based on the method of [9]), a FFR being a group of individuals that have haplotypes found co-occurring in heterozygotes. For this reason, the approach is not applicable on plastid or mitochondrial sequences. The FFR approach does not require monophyly and it was demonstrated that this method is among the best single-locus methods for delimitating species especially in species-poor data sets [10]. This performance is explained by the fact that construction of FFR relies on the verification of contemporaneous gene flow among the putative species, because gene flow is a crucial issue when one deals with species delimitation following the “biological species concept”. Biparentally inherited molecular markers have been widely used to estimate gene flow and to construct species phylogeny (e.g. [11–13]. This idea is reinforced by suggestion of a higher taxonomic value for nuclear markers in plants, because pollen dispersal contributes more to gene flow than seed dispersal (sole vector of gene flow for the plastid genome in most angiosperms) for most plant species [14].

Besides nuclear DNA sequences, widely used co-dominant markers such as nuclear microsatellites (or simple sequence repeats, SSRs) and single-nucleotide polymorphism loci (SNPs) are valuable to address population structure and species delineation in closely related taxa [15]. Assignment of genetic clusters to different species is automatic when the detected groups are found in sympatry and there is absence or scarcity of gene flow that cannot be explained by history (e.g. very recent secondary contact), ecological factors (e.g. microhabitats causing delay of phenology or existence of physical barriers) or particular breeding systems (e.g. autogamy, clonality). Guichoux et al. [16] suggested that microsatellites may be better than SNP to detect mixtures of genetic clusters (see also [17, 18]. But opposite findings have also been reported and the debate on the relative performance of SNPs over SSRs remains [19]. Ljungqvist et al. [20] suggested that using of five times more SNPs than SSRs are necessary to achieve the same discrimination power (see also [21, 22]). This ratio is not a rule but should depend on the characteristics of the loci used, the number of alleles, the degree of differentiation among the studied populations, and the methods used for marker development, which can at times generate ascertainment bias [23].

*Milicia* is an important African timber genus that has received attention from scientists during the last decade, although the focus was on populations found in Ghana and Cameroon (see a bibliography review in [24]). Phylogeographical and phylogenetical investigations by [25, 26] confirmed the existence of two morphologically similar species: *M. regia*, found West Africa from Senegal to Ghana, and *M. excelsa*, found from West to East Africa and part of Austral Africa. Several lines of evidence suggest that our current phylogeographic knowledge might be incomplete. First, relying on morphological characterization, the renowned botanist Auguste Chevalier claimed that *M. regia* naturally occurs in some parts of Gabon [27]. Second, East Africa was poorly represented in [26] while it might harbour genetically original populations, as reported in other widely distributed African plant species (e.g. [28, 29]). Third, the heterogeneous sampling intensity of the previous works might have affected the power to detect distinct genetic clusters [30] so that a more systematic sampling may reveal additional patterns. In addition, the degree of similarity or overlap of the environmental envelopes of the inferred genetic clusters as well as the degree of correlation between genetic distances and niche overlap measures have seldom been addressed in African plant taxa, although these issues may give hints on the relative importance of neutral *vs*. adaptive forces underlying genetic differentiation between clusters [31, 32].

The present study revisits the genetic diversity of *Milicia* populations in Africa along with an assessment of its relationships with climate niche patterns using a more homogeneous sampling. We compare the ability of SSRs and SNPs for delimiting genetic clusters and species, and detect any hidden genetic diversity in *Milicia* populations, and we use a nuclear DNA sequence to assess phylogenetic relationships. More specifically, we addressed the following questions: (1) Does *Milicia regia* naturally occur in Central Africa as reported a century ago [27], and if so, what is the degree of divergence between the populations of the genus *Milicia* in Central and East Africa? (2) What is the degree of congruence between the different genetic markers in terms of population structure and history? (3) Is there any sign indicating that habitat selection may have contributed to the genetic divergence among *Milicia* genetic clusters?

## Methods

### Study taxa, sampling context and sampling plan

The genus *Milicia* contains two species in sympatry in West Africa: *M. regia* and *M. excelsa*. Whereas the range of *M. regia* seems to be restricted to the Upper Guinean domain, *M. excelsa* stretches in various African forest types from West to East Africa. Both species are wind pollinated and seeds are dispersed by bats and parrots [25]. Genetic evidence showed that they constitute two reproductively isolated groups despite existence of paraphyly in *M. excelsa* [26]. In West Africa, *M. regia* is considered as an endangered species due to overexploitation for timber production and deforestation, and is listed as a vulnerable species by the IUCN [24]. There is no particular logging restriction regarding the widespread *M. excelsa* in Central Africa, except for a minimum cutting diameter.

**Fig. 1:**
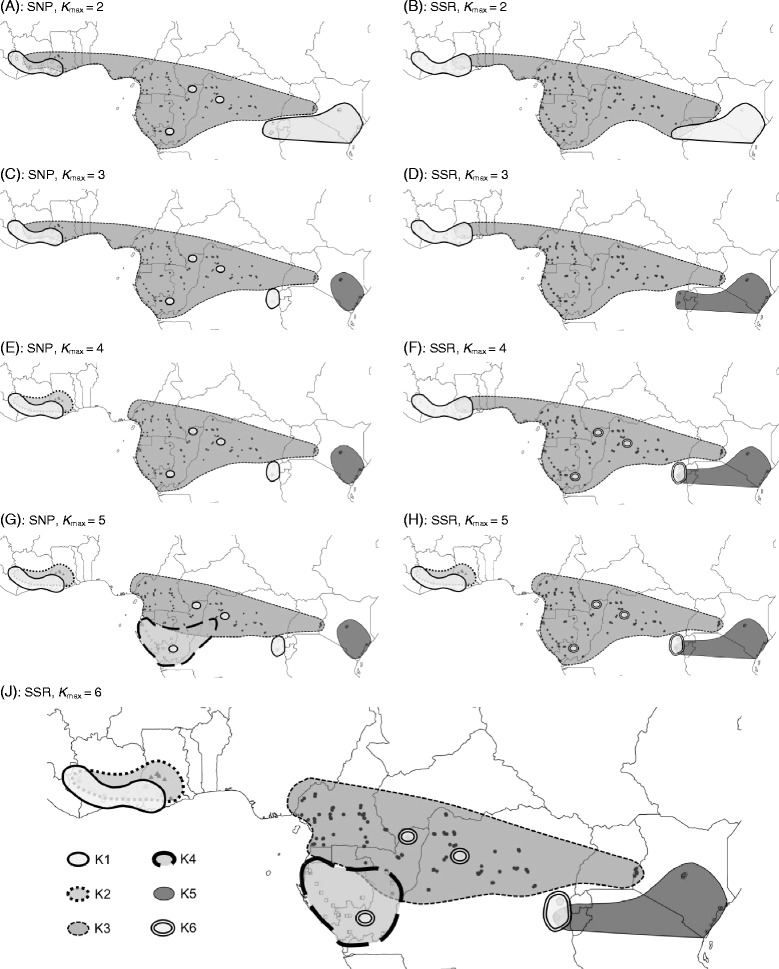
Evolution of genetic clustering among 435 *Milicia* samples according to *K*_max_. *K*_max_ was increased from 2 (**a**, **b**) to 5 (**g**, **h**) and 6 (**j**) using nuclear SNPs (left; **a**, **c**, **e** and **g**) or SSRs (right; **b**, **d**, **f** and **h**). **j** shows the most likely scenario with K = 6 genetic clusters according to SSRs genotypes. Each combination of grey tone and type of outline stands for a given genetic cluster

A project dedicated to wood traceability purposes has been recently achieved (ITTO Project PD 620/11 M (Rev. 1); [33]). One of its specific objectives was to document the genetic structuring in *Milicia* species at a higher spatial resolution than previous works that detected five geographically coherent genetic clusters [34, 35]). To this end, a systematic sampling was carried out in seven countries: Ivory Coast, Ghana, Cameroon, Gabon, Republic of the Congo, Democratic Republic of the Congo and Kenya, with 10-20 individuals sampled in grid-cells of 100-150 km side (depending on the country) laid out on the known range of the genus. For the present study we retained a subsample of 435 individuals distributed over the study zone (Fig. 1) which represented a substantial part of the range of the genus.

### Genotyping and sequencing

These 435 individuals were genotyped at seven nuclear microsatellites following [26] and at 67 biallelic nuclear SNP loci. These SNP markers were developed using an approach of the Thünen Institute for Forest Genetics (TIFG). The method is based on a restriction associated DNA sequencing protocol (RADseq). Two samples of *M. excelsa* from Benin and Kenya were used. Libraries were prepared using the restriction enzyme *Sbf*I, and sequenced on the Illumina HiSeq 2000 platform to create paired end reads of 2 x 100 bp. SNPs were identified in the sequenced individuals using variant call format (VCF) 4.1 (Floragenex). Marker screening was conducted in a sample comprising 95 individuals of *M. excelsa* and 16 individuals of *M. regia* in Assay Design Suite (ADS) (Agena Bioscience) and genotyped on the MassARRAY iPLEX platform (Agena Bioscience) (C. Blanc-Jolivet and B. Degen, in preparation). The same team of the TIFG contributed to the development of similar SNP markers in another African tree species in the framework of the aforementioned project and the protocol is described in [36]. SNPs have been chosen because they can be assayed using shorter DNA fragments than microsatellites, an advantage using degraded DNA extracted from wood. In a subset of 190 individuals, we further sequenced two intergenic regions of the plastid genome, *psbA-trnH* and *trnC-ycf6,* and one genic region of the nuclear genome, *At103*, the only polymorphic region observed among 12 tested gene regions in *Milicia* [37]. The protocol for sequencing is described in [26].

### Genetic structure of *Milicia* populations and morphological differentiation of genetic clusters

We ran TESS 2.3.1 [38] to identify genetic clusters separately using the SNPs and SSRs datasets. The protocol was as follows: the maximum number of clusters, *K*_max_, was fixed between 2 and 10; we chose an admixture model and the interaction parameter, *ψ*, was set to 0 (i.e. spatial information is not used to identify genetic clusters); each run consisted in 20,000 iterations with a 5000 burn-in period, and we performed 10 runs for each value of *K*_max_. Then for each type of markers, we plotted values of the deviance information criterion, DIC, against *K*_max_ to infer the likely number of clusters. The average cluster membership, *q*, of each individual was finally determined (program CLUMPP; [39]) and individuals were assigned to a given cluster when *q* > 0.5. Because both SNP and SSR loci detected a questionable genetic cluster of scattered but well delimited populations into the Central African region with genetic affinities with *M. regia*, the latter being expected only in West Africa (see results), we also verified the leaf morphology of samples from each genetic cluster under a stereomicroscope. The lower leaf surface of adult specimens of *M. excelsa* should present microscopic hairs in contrast to *M. regia* [26].

For each inferred genetic cluster, the following diversity parameters were computed using SPAGEDI 1.4 [40]: the effective number of alleles, *NAe* [41], the allelic richness, *AR* (for a given number of gene copies), and the gene diversity corrected for sample size, *He*. The degree of differentiation between genetic clusters was assessed through three parameters: *F*_ST_ and Nei’s standard genetic distance with sample size correction, *D*_S_, which are both allele identity based statistics, and (*δμ)* ^2^ based on microsatellite allele size (hence not computed for SNPs) [42]. *D*_S_ and (*δμ)* ^2^ are expected to better reflect divergence time than *F*_ST_, which depends much on genetic drift, and can be used for phylogenetic reconstruction.

### Phylogeny and estimate of divergence time among genetic clusters

Microsatellites can be useful in phylogenetic reconstruction under the stepwise mutation model and mutation-drift balance (e.g., [42, 43]) even for taxa that have diverged as long as 30 mya [44]. Therefore, we verified whether the SSRs-based phylogeny of genetic clusters could confirm the phylogenies inferred from DNA sequences (*psbA-trnH*, *trnC-ycf6,* and *At103*). Using POPTREE 2 and following [45], we constructed phylogenetic trees based on *D*_S_ and (*δμ)* ^2^ computed from the SSRs data. Trees were constructed with the Neighbor-Joining method and tree validity was evaluated by bootstrapping (10,000 replications). We also estimated divergence time *t* between pairs of genetic clusters via the equation *(δμ)* ^2^ = 2*μG* [46], with *μ* being the mutation rate per locus per generation and *G* the number of generations after the divergence of the two considered populations. We assumed 100 years per generation and *μ* ranging between 5.0 x 10^-6^ and 10^-3^ per generation per locus for microsatellite loci [47, 48].

For nucleotide sequence data, we first constructed a median joining network for each sequence using NETWORK 4.6 [49]. Thereafter only the nuclear sequence *At103* was employed for further analyses as plastid sequence data provided poor separation of the different genetic clusters (see results). Haplotypes were reconstructed with PHASE implemented in DnaSP [50] and CHAMPURU [51] for length variant heterozygotes. A haploweb *sensu* [8] was constructed by connecting haplotypes occurring in heterozygous individuals in order to identify fields for recombination (FFR). To verify congruence between SSRs data and those of the nuclear sequence *At103*, we also constructed a phylogenetic tree based on *At103* haplotypes through the Bayesian method implemented in *BEAST [52]. From a previous analysis in [26], we dated the ancestor of *Milicia* at 8-41 mya (95% posterior estimate of the age distribution) and this was utilized as a prior for the analysis. A Yule tree prior and an uncorrelated relaxed molecular clock were assumed. The MCMC was run seven times for 500 million generations, each run being sampled every 50,000 generations, and the final tree had the highest posterior probabilities at the nodes.

### Modelling the environmental niche of genetic clusters and evaluating correlation between niche overlap and genetic distance

First, we inferred putative geographical locations of each genetic cluster detected with TESS 2.3.1 [38] through environmental niche modelling using Maxent [53] with the logistic methods and the default settings for the maximum entropy. Last Glacial Maximum (LGM, 21,000 years ago) climatic variables obtained at 2.5 arc-minute resolution from the WorldClim global dataset [54] were considered for niche modelling. Principal component analysis (PCA) was used as a data reduction technique to avoid model over-fitting linked to correlated predictor variables [55]. We retained a 500 km-buffer zone to the whole dataset in order to reach the known limits of *Milicia* range in Africa and because this gave the best models based on preliminary values of the Area Under the Curve (AUC; from 0.89 to 0.97). Analyses were performed with the R environment [56].

Second, we applied a smoothing technique through a PCA that divides the environmental space (delimited by the minimum and the maximum climatic values) in cells and uses a kernel function to determine the smoothed density of occurrence for each genetic cluster to each cell *i* (100-km^2^ each) [57]. Then we computed the metric *D* developed by [58] for pairwise niche overlap (*D* = 1 – 0.5 ∑ |*p*_X,i_ - *p*_*Y*,i_| where *p*_X,i_ and *p*_*Y*,i_ stand for the probability assigned by the ENM (Environmental Niche Modelling) for genetic clusters *X* and *Y*, respectively, to cell *i*). The statistic *D* varies from 0 (no overlap between the two considered niches) to 1 (the two niches are identical). Finally, the correlation between genetic distances as expressed by *D*_S_ or (*δμ)* ^2^ from SSRs and niche overlap measure *D*, was tested by the means of a simple Mantel test in order to verify whether niche overlap was higher – or not – in genetically similar clusters. Recent studies revealed that genetic divergence of specific gene markers can be a good predictor of differentiation at quantitative trait loci in random mating populations [31]. That is, divergence in neutral loci can reflect adaptive phenotypic selection (reviewed in [32]). Hence any significant correlation between *D* and *D*_S_ or *(δμ)* ^2^ may reflect a genetic signature of divergent selection across some genetic clusters of *Milicia,* especially those in the Congo Basin where climate is quite similar across a large region. We chose genetic divergence measures from SSRs because they are superior at elucidating the genetic structure of *Milicia* populations (results). A total of 10,000 permutations were performed for testing the significance of the Mantel tests.

## Results

### Genetic clustering from SSRs vs SNPs: evidence of a closely related taxon of *M. regia* in Central Africa

When applying genetic clustering using the SNP dataset the increment of the likelihood of the data with *K*_max_ displayed a steep positive slope to *K*_max_ = 4 followed by a shallower positive slope with *K*_max_ without an asymptotic trend (Additional file 1: Figure S1). Using the SSR dataset the substantial increase of likelihood occurs up to *K*_max_ = 6 where an asymptote seems to be reached (Additional file 1: Figure S2). The clustering patterns defined geographically coherent genetic clusters (with one exception discussed later) and were globally congruent between SNPs and SSRs, except that the order at which the genetic clusters appeared as *K*_max_ increased differed between types of markers (Fig. 1). SNPs and SSRs clustering patterns were globally congruent when *K*_max_ = 5 for SNPs (Fig. 1g) and *K*_max_ = 6 for SSRs (Fig. 1j). The main difference is that one of the SNPs genetic clusters was divided into two clusters according to SSRs: one in West Africa, K1, and another one in Central Africa, K6.

The West African individuals of *M. regia* formed one genetic cluster named K1. West African individuals of *M. excelsa* formed another cluster named K2. Both types of markers also identified a large genetic cluster of *M. excelsa*, K3, stretching from the forest zone of Cameroon to the Northern parts of the Republic of the Congo (RC) and Democratic Republic of the Congo (DRC). A genetic cluster K4 covered the Western part of Gabon and appeared only at *K*_max_ = 5 for the SNPs and *K*_max_ = 6 for the SSRs (Fig. 1). A fifth genetic cluster, K5, covered Kenya (and the East of DRC based on the SSR dataset). The main difference between the two types of markers was the finding of 13 Central African individuals that were assigned to cluster K1 (*M. regia*) according to SNPs (Fig. 1g) whereas SSRs grouped 12 of these individuals in a new genetic cluster that we named K6 (Fig. 1j; Table 1). Contrary to the other clusters which display distinct distributions, K6 displays a disjoint spatial distribution largely embedded in the range of clusters K3 and K4.

**Table 1:** Number of individuals assigned to each cluster at *q* = 0.50 for SNP and SSR analyses. Numbers in bold in the diagonal indicated individuals jointly assigned by SNPs and SSRs to a same genetic cluster

	SSRs genetic clusters								
		K1	K2	K3	K4	K5	K6	Undefined	Total
SNPs genetic clusters	K1	**44**	4	3		1		10	62
	K2	1	**30**	4				2	37
	K3			**197**	17	3		2	219
	K4			5	**67**				72
	K5			1		**30**			31
	K6						**12**	1	13
	Undefined							**1**	1
	Total	45	34	210	84	34	12	16	**435**

Regarding the order of appearance of genetic clusters as *K*_max_ increases, according to SNPs, the Central African assigned-to-K1 individuals appeared as soon as *K*_max_ was fixed to 2, whereas these same individuals (with the exception of one tree) were isolated from the largest Central African cluster only at *K*_max_ = 4 according to SSRs (Fig. 1). Another important difference came from the genetic cluster K2 grouping West African *M. excelsa*: it was detected at *K*_max_ = 4 with the SNPs, and at *K*_max_ = 5 with the SSRs. Both types of markers grouped Kenyan individuals with West African *M. regia* individuals at *K*_max_ = 2, but distinguished them at *K*_max_ = 3. SNPs and SSRs detected the Gabonese cluster K4 only at their final respective scenario (5 clusters for SNPs and 6 for SSRs). As the most questionable genetic cluster was K6 given its disjoint distribution and its inclusion in K1 (*M. regia*) according to the SNPs, we verified the morphology of all individuals from that genetic cluster and samples from the neighbouring genetic clusters. Our observations confirmed that all the 13 individuals identified as *M. regia* according to the SNP display the specific leaf feature of *M. regia* (Additional file 1: Figure S3). Individuals in genetic clusters K2 to K5 harbour microscopic hairs characteristic of *M. excelsa*.

**Fig. 2:**
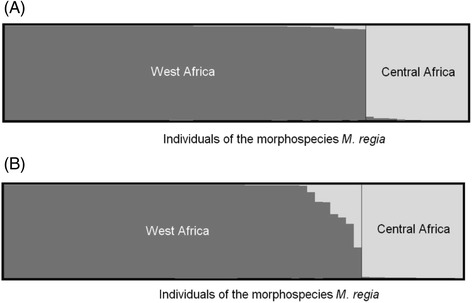
Membership coefficient of individuals that present leaves of *M. regia*. **a** was based on SSRs whereas **b** was based on SNPs. *K*_max_ = 2 was considered for both figures

Finally, when using TESS to evaluate the genetic structure among the *M. regia* genetic clusters, K1 of West Africa and K6 of Central Africa, SNPs and SSRs markers were congruent, identifying two clusters (Fig. 2), with no evidence of admixture according to SSRs, in contrast to the results from the SNPs (bias likely due to the low diversity of SNPs in *M. regia*, and thus limited information content to distinguish genetic clusters) (Fig. 2). Even when we considered only Central African clusters of *Milicia*, there was no evidence of substantial gene flow between K6 and the surrounding populations (Additional file 1: Figure S4). Hence even for the SNPs, we will consider thereafter morphological *M. regia* individuals of Central Africa as forming a sixth genetic cluster.

### Congruence of SNPs and SSRs in estimating diversity and differentiation parameters

A total of 380 of the 435 individuals analysed were jointly assigned to the same genetic clusters by both types of markers, making an average concordance of 87% (Table 1). In order to reliably compare SSRs and SNPs we considered only these 380 individuals for the estimates of diversity and differentiation parameters. SSR loci provided a total of 65 alleles whereas the 67 biallelic SNP loci provided 134 alleles (allele ratio SNP/SSR = 2.06). Values of diversity parameters tend to be lower with the SNPs than the SSRs due to the biallelic state of the SNPs. The trends among types of markers were similar if we consider only genetic clusters of the morphospecies *M. excelsa*: the Kenyan cluster, K5, displayed the lowest values whereas genetic diversity was in the same order for the other clusters K2, K3 and K4. Markers differed strikingly when comparing the two species: SSR data provided the highest diversity values for the West African *M. regia* genetic cluster, K1, while SNPs displayed the opposite trend, with the clusters of *M. excelsa* exhibiting the highest diversity values (Table 2). The proportion of polymorphic SNP loci in *M. excelsa* was 100% whereas it reached 72 and 22% in *M. regia* genetic clusters K1 and K6, respectively (Table 2; Additional file 1: Table S1).

**Table 2:** Diversity parameters among the six inferred genetic clusters in *Milicia* populations. *NAe* effective number of alleles, *AR* allelic richness, and *He* gene diversity corrected for sample size, *Npl* proportion of polymorphic SNP loci

Genetic clusters	Sample size	SSRs			SNPs			
		*NAe*	*AR*	*He*	*NAe*	*AR*	*He*	*Npl*
K1	44	3.93	2.71	0.7243	1.08	1.06	0.0632	0.72
K2	30	2.33	2.12	0.5372	1.37	1.23	0.2283	0.87
K3	197	2.07	1.98	0.4794	1.56	1.33	0.3275	0.99
K4	67	2.03	1.89	0.4405	1.46	1.27	0.2744	0.91
K5	30	1.71	1.68	0.3181	1.16	1.12	0.1175	0.82
K6	12	1.98	1.88	0.4205	1.04	1.03	0.0297	0.22

**Fig. 3:**
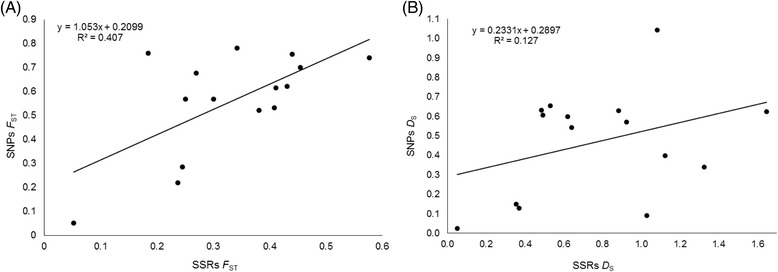
Correlation between genetic differentiation estimates from SNPs and SSRs in *Milicia* genetic clusters. **a** was based on *F*ST; **b** was based on *D*_S_

There were also significant differences between SNPs and SSRs in estimating degree of genetic divergence in pairs of genetic clusters. For *F*_ST_, the values calculated from SNPs were higher than those from SSRs (Wilcoxon Matched Pairs test; *Z* = 2.38, *P* = 0.017); that trend was reversed if we considered *D*_S_ (Wilcoxon test; *Z* = 3.24, *P* = 0.001). As expected from divergent evolution in the clustering pattern (Fig. 1), the two measures of genetic differentiation between clusters were not strongly correlated with a coefficient of determination *R* ^*2*^ = 0.13 for *D*_S_ and *R* ^*2*^ = 0.41 for *F*_ST_ (Fig. 3). There was nonzero y-intercept (Fig. 3). Finally, we considered the pair of *M. regia* genetic clusters, K1 and K6 to compare *F*_ST_ and *D*_S_ (Fig. 2). Whereas SNPs *F*_ST_ reached 0.57 for that pair (the global pairwise *F*_ST_ was 0.56), *D*_S_ for the same pair of genetic clusters was 0.09 (the global pairwise *D*_S_ of 0.47) (Table 3). In general *D*_S_ better reflected the clustering pattern and was better correlated to *(δμ)* ^2^ in microsatellites than did *F*_ST_ (Pearson’s *R* = 0.74 for *D*_S_-*(δμ)* ^2^ vs. R = 0.55 for *F*_ST_-*(δμ)* ^2^ (from data in Table 3).

**Table 3:** Estimates of genetic distances and niche overlap measure (*D*) between *Milicia* genetic clusters. The degree of genetic differentiation was based on *F*_ST_, Nei’s *D*_S_ and Goldtstein’s *δμ* ^2^ computed from genotypes at SNP and SSR datasets

Genetic clusters		SSRs			SNPs		Niche overlap (*D*)
*X*	*Y*	*δμ* ^2^	*F*_*st*_	*D*_s_	*F*_*st*_	*D*_*s*_	
K1	K2	2.726	0.185	0.486	0.759	0.632	0.180
K1	K3	3.030	0.250	0.491	0.569	0.608	0.292
K1	K4	4.328	0.270	0.531	0.677	0.655	0.303
K1	K5	3.892	0.343	1.122	0.782	0.398	0.479
K1	K6	10.795	0.301	1.030	0.568	0.091	0.046
K2	K3	2.231	0.237	0.370	0.220	0.130	0.129
K2	K4	2.876	0.245	0.356	0.286	0.150	0.078
K2	K5	7.193	0.440	1.081	0.756	1.043	0.060
K2	K6	14.100	0.455	1.647	0.700	0.624	0.114
K3	K4	0.351	0.052	0.049	0.051	0.024	0.475
K3	K5	4.758	0.381	0.641	0.522	0.543	0.280
K3	K6	8.026	0.409	0.924	0.533	0.570	0.200
K4	K5	6.587	0.411	0.619	0.616	0.599	0.237
K4	K6	9.800	0.431	0.882	0.622	0.631	0.068
K5	K6	8.624	0.577	1.324	0.740	0.340	0.070
Global pairwise genetic distance		-	0.333	0.771	0.560	0.469	-

### Phylogenetic reconstruction in *Milicia* genetic clusters

For the two chloroplast regions, *psbA-trnH* and *trnC-ycf6*, only the *M. regia* genetic cluster in West Africa (K1) harboured specific haplotypes. Individuals of the cluster K6 shared their haplotypes with the other *M. excelsa* populations (Additional file 1: Figures S5 and S6).

**Fig. 4:**
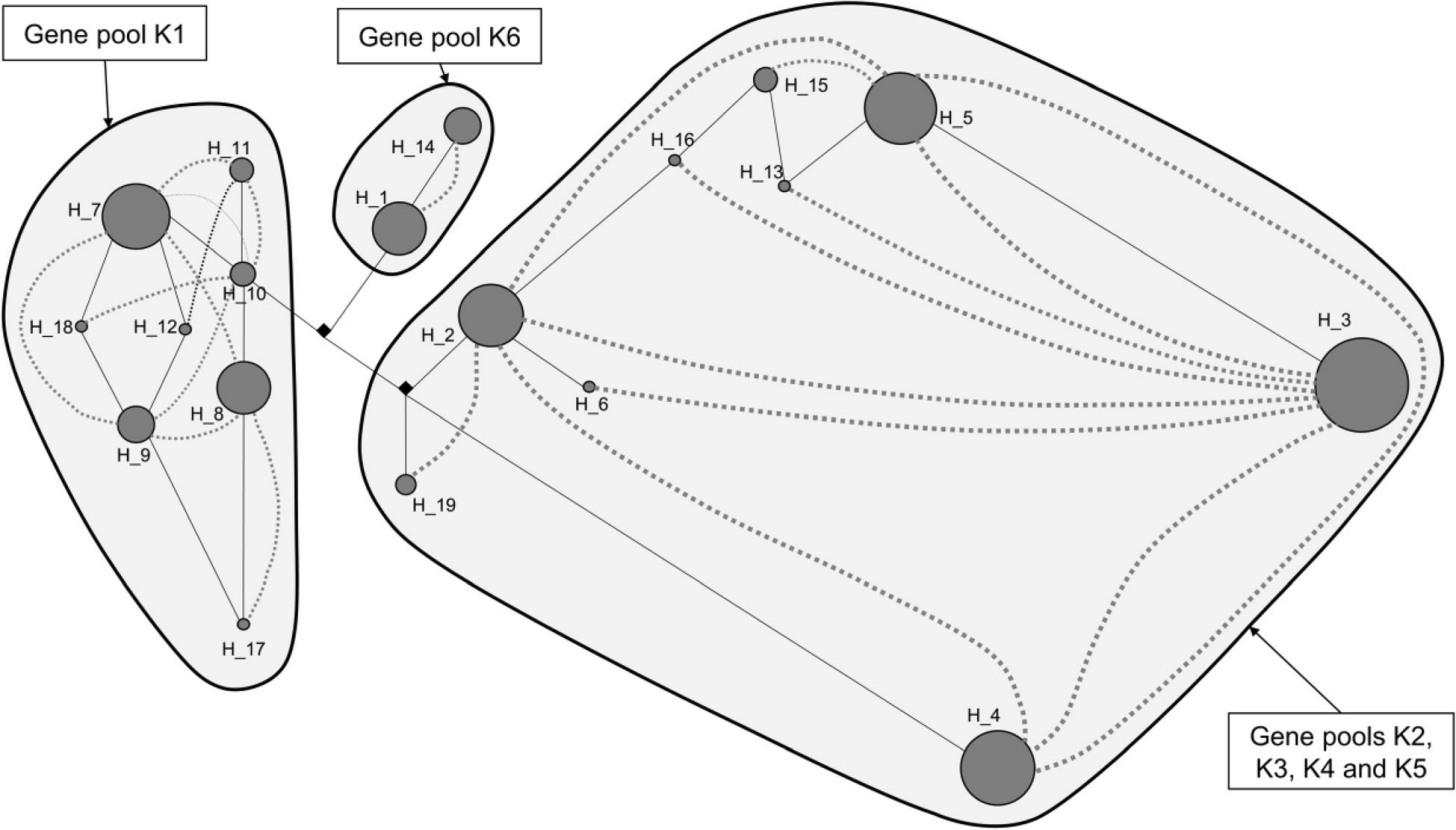
*At103* haplotype network and haploweb (*sensu* [8]) in the genus *Milicia*. Circles that stand for each haplotype (H_1, H_2, etc.) are proportional to the number of individuals. The length between a pair of haplotypes is proportional to the number of mutations separating them. *Dashed curves* link together haplotypes of heterozygous individuals. Each surrounded group of haplotypes indicates a single-locus field for recombination (FFR) and the corresponding genetic clusters (K1 to K6) are mentioned beside. Individuals of the morphospecies *M. regia* are exclusively found in two genetic clusters, K1 (West African trees) and K6 (Central African trees)

The nuclear sequence *At103* from 130 individuals exhibited a different pattern. First, individuals of the morphospecies *M. regia* found in central Africa (cluster K6) presented specific haplotypes, H_1 and H_14 (Fig. 4). Determination of FFR through haploweb construction confirmed that contemporaneous mating does not occur between these Central African individuals of *M. regia* and any other group of *Milicia* individuals (Fig. 4). *M. regia* in West Africa (genetic cluster K1) also displayed a specific FFR with eight haplotypes whereas *M. excelsa* populations from West and Central Africa (genetic clusters K2, K3, K4 and K5) shared a unique FFR (Fig. 4).

**Fig. 5:**
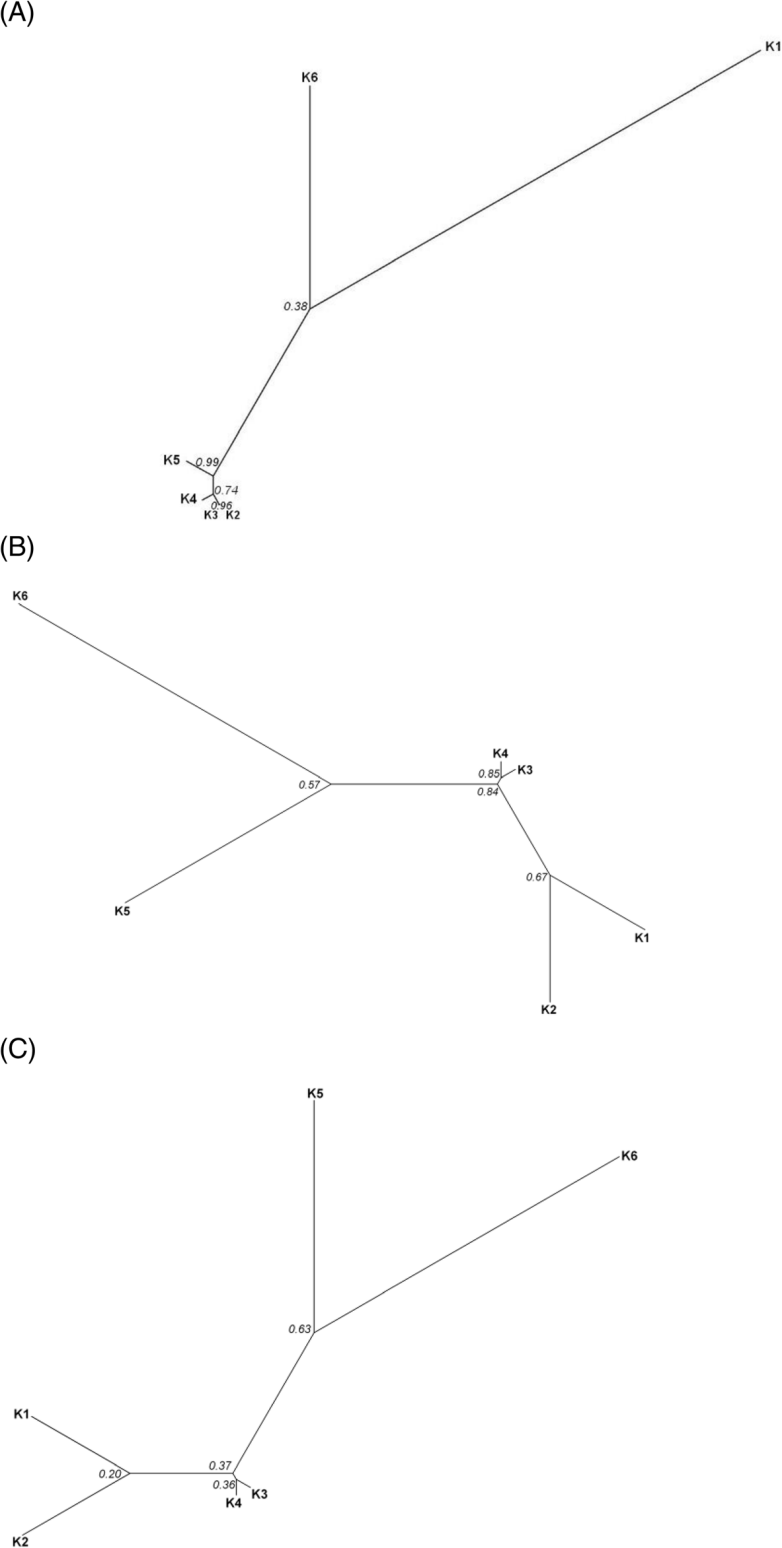
Phylogenetic trees from *Milicia* genetic clusters. The trees were constructed from *At103* sequences (**a**), genetic distances *D*_S_ based on nuclear microsatellite genotypes (**b**) and SNPs dataset (**c**) considering the genetic clusters (K1 to K6). Italic number at the nodes indicate posterior probabilities (**a**) or bootstrap values (**b** and **c**)

In terms of phylogenetic relationships, analyses of the nuclear sequence *At103* showed that the clusters K6 and K1 (morphologically *M. regia*) tend to depart from the other genetic clusters (*M. excelsa*) but with a weak support (0.38; Fig. 5a). The most recent common ancestor of all genetic clusters was dated at 8.71 mya (range of 0.15 to 35.25 mya, with a confidence interval of 95%). Both phylogenetic trees from microsatellite and SNPs data based on *D*_S_ exhibited similar trends and contrasted with the *At103* scenario on one point: the relative position of the *M. regia* cluster K1 that showed more affinity with the genetic cluster K2 than with the conspecific cluster K6 (Figs. 5b and c). The three phylogenetic trees agreed to identify the cluster K6 as the most divergent group. With microsatellite loci, the most ancient split was detected between K2 and K6, dated between 0.07 – 1.41 mya.

### Niche overlap between *Milicia* genetic clusters and correlation with genetic distance

The occurrence range of the six genetic clusters was well explained by the following climatic variables correlated to the first PCA axis (68.5% of the total variance): annual mean precipitation, annual mean temperature, annual temperature range, solar radiation, and precipitation of the driest quarter (Additional file 1: Figure S7). The niche model map of each genetic cluster is presented in the Additional file 1: Figure S8. Globally, niche overlap values were low, ranging between 0.060 (K2-K5) and 0.475 (K3-K4) (Table 3). The Mantel test between niche overlap values *D* and the genetic distance *D*_S_ resulted in a regression slope *b* = -0.131 (*R* = -0.39) which was not significant (*P* = 0.159). A similar analysis using *D* and *(δμ)* ^2^ resulted in *b* = -0.022 (*R* = -0.581) which was significant (*P* = 0.016).

## Discussion

According to the sampling scheme and the markers used genetic studies can either detect or miss hidden genetic diversity. In particular the sampling approach may be a major issue. Daïnou et al. [26] did not highlight any particular genetic species specificity from a sample of 849 individuals of *Milicia* because their sampling was not spatially regular (overrepresentation of some locations). Owing to new populations included in the analyses and a more homogeneous geographic sampling with a lower number of individuals (535 individuals), we showed that both SSRs and a few dozens of SNPs are good marker candidates to reliably characterize the genetic structure within a taxon.

As hypothesized, East African populations of *Milicia excelsa* strongly diverge from the Central and West African populations, a pattern found in other species [28, 59], and mirrored by the clear differentiation of the East Africa flora compared to the remainder of the continent (e.g. [60]). But the most important finding came from the *Milicia* genetic cluster K6 made of scattered Central African samples morphologically similar to *M. regia*. This species is known to occur only in West Africa westwards of Togo, hence one may think that these individuals could be remnants of historical plantations of *M. regia* (as supported by SNPs). However, we found no report of such plantations, while we rediscovered a century-old article reporting *M. regia* in Gabon [27]. Further investigations using other markers confirmed that the morphospecies *M. regia* observed in Central Africa is strongly divergent from the other populations of *Milicia*: (i) the clustering pattern from SSRs that considered this group as a different genetic cluster; (ii) the absence of gene flow with the other clusters; and (iii) the haploweb outputs from the nuclear sequence *At103* that identified all these K6-individuals as a separate field for recombination. Furthermore, phylogenetic reconstructions suggested that the genetic cluster K6 is probably as old as the ancestors of all *Milicia* populations. Divergence among *Milicia* genetic clusters looks to have been shaped by geographic isolation probably in relation to past ice ages but there was also a signal of habitat selection effect (significant correlation between niche overlap and the genetic distance *(δμ)* ^2^).

### Discovering hidden genetic diversity: beyond the sampling scheme are the type of markers and the analytical tools

In case of weak morphological differentiation among taxa the discovery of cryptic species is most of the time a matter of chance [61], unless there is some observation-based evidence of lack of mating between the sibling groups (e.g., [62, 63]). At the beginning of the 21^st^ century, barcoding techniques were used to detect hidden genetic diversity in the form of two or more phylogenetically distinct clades corresponding to slightly different phenotypic groups or having distinct geographical distributions [6, 64]. The advantage of sequence data is that they require a low sampling density although it has been criticized [65]. Detection of polyphyletic patterns may only be conclusive by maximizing the number of samples per geographical location and the number of places for collection. The major limit of phylogenetic approaches based on sequence data in addressing cryptic species issues is that the observation of paraphyly or polyphyly does not allow to identify species although reproductively isolated groups may exist. In the absence of population genetics data additional information such as allopatric distribution, substantial differences in morphology (preferably qualitative characters; [7]) or any other observations suggesting mating barrier may be necessary to argue for the presence of cryptic species [6, 66–68]. As a consequence the haploweb approach looks as a good alternative for species delimitation.

Species delimitation via haplowebs has been proved to be better than coalescent approaches or gap detection method in species-poor data sets (one to three species; [10]). However, haplowebs can also provide biased conclusions when population sizes and speciation rates are large [10]. *Milicia* is not a young genus [26] and as it is known to contain only a few species (two species before the present study), we can argue that rapid radiation is not relevant here and should not affect performance of haploweb. But dense sampling can be a concern: more heterozygous individuals may contain rare shared alleles which may obscure the global pattern, leading to underestimation of the true number of species. With the exception of the cluster K6, sample size per genetic cluster was quite high in the present study ranging from 30 to 197. That putative problem can be solved by using several independent markers for constructing the haplowebs, but this was not possible in our case because only one of the 12 tested nuclear sequences was polymorphic. Specific haplotypes from the two chloroplast sequences were found for only one genetic cluster: the West African populations of *M. regia*. Although not employed as much as *trnH-psbA*, *trnC-ycf6* got a certain success when combined with the former (e.g., [69]). *trnH-psbA* is probably the most used plastid intergenic barcode after *rbcL + matK* [70] and shows good species identification success rates [71] including in Moraceae such as *Ficus* [72]. Therefore it can be useful when aiming at revealing hidden species diversity (e.g. [73]). But it failed in the case of *Milicia*.

### *Milicia* evolutionary history and incongruence between gene genealogies

Haplotype sharing between the cluster K6 and *M. excelsa* individuals from chloroplast sequences may suggest either a strong relatedness between those populations along with incomplete lineage sorting, or past chloroplast capture. If we remove from consideration the West African cluster of *M. regia* K1, the divergence time of K6 and its relatedness to Kenyan cluster K5 composed of *M. excelsa* (Fig. 5b and c) supported the first hypothesis as this phenomenon is quite common in recently diverging species with large effective population size [74]. The chloroplast capture scenario is also acceptable. It is a common phenomenon between closely related plant species, and there are already several examples that are explained by such events (e.g. [75]). Theory predicts that when a species extends into the range of a related species that can occasionally hybridize, a hybridisation event followed by recurrent backcrosses can lead to the capture of the chloroplast of the local species by the invading one [76]. We can thus hypothesise that this had happened in the past when ancestors of K6 penetrated the range of *M. excelsa* in Central Africa. Additional investigations with new markers could help to identify the best scenario.

Niche modelling techniques offer a good way to verify relationships between population genetic divergence and environmental selection. Daïnou et al. [26] already developed a scenario on the possible impact of past climate changes on population demography in *Milicia*. The Mantel test between *(δμ)* ^2^ and niche overlap *D* performed in the present study resulted in a substantial and significant correlation (*R* = -0.58). This should reflect signs of selection acting for the differentiation between the genetic clusters of *Milicia,* even at intraspecific level for *M. excelsa* [32], and this took place many thousands of years before as the modelling of niches was based on climatic data from the Last Glacial Maximum (≈20,000 BP). We need to moderate the value of the correlation as the outcomes of niche modelling for some *Milicia* genetic clusters could be unreliable or incomplete. Indeed, the outputs of those approaches, especially Maxent technique, can be biased by samples provided for the modelling [77]. The West African samples implemented here in the environmental modelling was poor as it covered only a few countries whereas the genus occurs from Senegal to Nigeria in that region. Therefore, we do think that further investigations related to niche characteristics should be conducted later in order to better assess signs of any putative selection effect on genetic cluster differentiation.

### SNPs vs SSRs: high congruence for the contemporaneous genetic structure but divergent histories

Due to the biallelic character of most of SNPs these markers are usually considered as less informative than polymorphic microsatellites to highlight a genetic structure, for a similar number of loci. Hess et al. [22] found that SNP loci may require 8-15 times the number of SSR loci to delineate with equivalent power a mixture of individuals from differentiated populations (see also [78]). As our number of SNP loci was 9.6 times the number of microsatellites markers, we could thus expect similar power. It is probably more relevant to compare the total number of alleles minus the number of loci between the two set of markers, which gives 134-67 = 67 for SNPs and 65-7 = 58 for SSRs. Thus, there would be a slight advantage for our set of SNPs. Accordingly, in West Africa, SNPs performed well to delineate the two species whereas SSRs exhibited a substantial proportion of putatively admixed individuals that may reflect a more limited power of SSRs to separate species, unless hybridization is more pronounced than assumed between *M. excelsa* and *M. regia* in West Africa (SSRs better detect admixed individuals; [16]). However, SNPs systematically merged K6-individuals with West African *M. regia* individuals up to *K*_max_ = 7 (not shown; signs of separation between K6 and K1 appeared at *K*_max_ = 8). As the clustering solution of SSRs was clearly supported by the *At103* sequences that demonstrated that K6 bears exclusive haplotypes, SNPs appeared less powerful than SSRs to discriminate genetic clusters in Central Africa.

Another important difference between SSRs and SNPs was observed in the trend of genetic diversity among genetic clusters for each type of marker. Whereas SSRs exhibited the highest sequence diversity in the West African *M. regia* cluster K1 (*He* = 0.72 compared to *He* in the range 0.32 to 0.54 for the other genetic clusters), SNPs displayed much lower diversity values in both *M. regia* clusters K1 (*He* = 0.06) and K6 (*He* = 0.03) as compared to *M. excelsa* genetic clusters (*He* in the range 0.12 to 0.33). Ascertainment biases due to marker discovery protocols can explain those differences. In microsatellites, the hypothesis of length ascertainment bias states that the median or mean allele size of microsatellites is the greatest in the species or population that has served for the development of the markers. Homologous loci in sister species may have different evolutionary histories so that a locus characterized in a sister species may not be as polymorphic as in the one from which SSRs have been derived [79]. In the present case, the SSR markers have been identified from a *Milicia excelsa* individual (sampled in the area of K2) and their polymorphism was evaluated in a sample composed of 30 trees of *M. excelsa* and 10 of *M. regia* from Ghana [80]. First, only three of the used SSR loci displayed a mean higher allele size in K2 comparatively to the other genetic clusters. Second, as the highest SSR diversity was not found in the cluster K2, there is no evidence of ascertainment bias in our SSR dataset. In fact, due to their high mutation rates, SSRs tend to be buffered from ascertainment bias comparatively to SNPs [23]. The SNP markers used in the present work have been identified from two *M. excelsa* individuals from K2 and K5, and the step of polymorphism screening for final marker selection involved only 17% of West African *M. regia* trees (C. Blanc-Jolivet and B. Degen, in preparation). As by definition SNPs are identified based on their polymorphism in the initially screened samples, ascertainment bias can be strong and this likely explains the much lower genetic diversity recorded in *M. regia* populations. As a SNP generally results from a unique mutation event and SNPs were assessed between the *M. excelsa* populations K2 and K5, only polymorphisms that appeared before the differentiation between *M. excelsa* and *M. regia* could remain polymorphic in both species. A comparison of SNP loci in morphologically assigned *M. regia* genetic clusters showed that among the 48 loci which were polymorphic in K1, only 14 were also polymorphic in K6. Only one locus was found polymorphic in K6 and not in K1. This clear ascertainment bias highlights that particular care should be made for the selection of SNPs for genetic structure characterization and that starting from a broad genetic basis is preferable.

## Conclusions

The present work highlights the value of large-scale genotyping of genera to discover cryptic species as well as highlight their hidden diversity at the intra-specific level. It is notable that, for a well-known timber tree, the occurrence of two species in Central Africa was not reported by botanists for a century although diagnostic leaf characters were known. Additional morphological investigations are required to evaluate at which extent the Central African new species of *Milicia* phenotypically resemble to the other species. In particular, floral and fruit characters should be meticulously examined. Additional file 1: Table S2 provides a list of individuals that were identified a priori in this new species, taking into account the entire sample of the ITTO Project. Because our sampling was not performed in a way that rare hybrids would be detected, next samplings should target the contact zones between the three species in order to verify more thoroughly any interspecific hybridization pattern.

We suspect that many similar cases remain, and that the floristic diversity of tropical forests remains underestimated. Recognizing cryptic species is particularly important for exploited species, like timber trees, as some of them might be endangered and require a special management policy while they are currently confused with a less vulnerable species. To identify cryptic species we showed that nuclear SNPs and SSRs can both be utilized and show similar resolution, while plastid markers are less reliable, a problem for current DNA barcoding in plants based on *rbcL* + *matK* sequencing. However, SNPs are prone to ascertainment bias than SSRs, at least when assessing genetic diversity, so that their development should ideally start from a large sample size. We recommend to collect and genotype hundreds of samples covering the distribution range of the taxon investigated.

## Additional file

Additional file 1: Table S1. Polymorphic (1) and monomorphic (0) SNP loci (P0040, P0095, etc.) in the six genetic clusters of *Milicia* (K1 to K6). **Table S2.** Geographic coordinates of Central African individuals of *Milicia* that may represent a new species according to SNP and based on the whole *Milicia* sample of the ITTO Project PD 620/11 Rev.1 (M): “Development and implementation of species identification and timber tracking in Africa with DNA fingerprints and stable isotopes” (1833 individuals). Individuals in bold are those selected for the SNP-SSR comparison (435 individuals in total) and which all turned out to be representatives of a new species as the nuclear gene *At103* confirmed. **Figure S1.** Determination of the number of genetic clusters in *Milicia* populations based on SNPs genotypes. **Figure S2.** Determination of the number of clusters in *Milicia* populations based on SSRs genotypes. **Figure S3.** (A) Lower surface of leaf for a tree morphologically identified as *M. regia* in Central Africa and confirmed by molecular markers, compared to a neighbour tree with typical leaves of *M. excelsa* (B). **Figure S4.** Graphical membership coefficient of Central African individuals of *Milicia* (genetic clusters K3 to K6) based on nuclear SSR loci. The six colours (blue, black, yellow, pink, green and red) stand for the six genetic clusters. The West African genetic clusters, K1 and K2, were not illustrated but were represented in some individual genome (blue and dark colors). **Figure S5.** Haplotype network and geographical distribution of *trnH-psbA* haplotypes in *Milicia* populations. **Figure S6.** Haplotype network and geographical distribution of *trnC-ycf6* haplotypes in *Milicia* populations. **Figure S7.** Ecological niches of the six genetic clusters of *Milicia* derived from nuclear SSR loci, in the environmental space produced by the principal component analysis method (PCA). The figure above indicates projection of the climatic variables in the plan formed by the two first axes (71.9% of the total variance). Similarly, the grey-to-black shading in the six small figures (1 = cluster K1; 2 = cluster K2; etc.) represents the grid cell density (black being the highest density) of the concerned genetic cluster in the PCA plan. The first dashed curve represents the 50% of the available environment space whereas the solid line stands for the entirety of the species environment. **Figure S8.** Potential distribution range of *Milicia* genetic clusters during the Last Glacial Maximum (LGM) according to niche modelling through Maxent approach. Black areas represent predicted regions with a probability higher than 50%. (DOCX 1353 kb)
