# Supplementary material for: Revealing hidden species diversity in closely related species using nuclear SNPs, SSRs and DNA sequences – a case study in the tree genus Milicia
Source: BMC Evol Biol. 2016 Dec 1;16:259. doi: 10.1186/s12862-016-0831-9 (PMC5131513; doi:10.1186/s12862-016-0831-9)
Supplement: Additional file 1: Table S1. — Polymorphic (1) and monomorphic (0) SNP loci (P0040, P0095, etc.) in the six genetic clusters of Milicia (K1 to K6). Table S2. Geographic coordinates of Central African individuals of Milicia that may represent a new species according to SNP and based on the whole Milicia sample of the ITTO Project PD 620/11 Rev.1 (M): “Development and implementation of species identification and timber tracking in Africa with DNA fingerprints and stable isotopes” (1833 individuals). Individuals in bold are those selected for the SNP-SSR comparison (435 individuals in total) and which all turned out to be representatives of a new species as the nuclear gene At103 confirmed. Figure S1. Determination of the number of genetic clusters in Milicia populations based on SNPs genotypes. Figure S2. Determination of the number of clusters in Milicia populations based on SSRs genotypes. Figure S3. (A) Lower surface of leaf for a tree morphologically identified as M. regia in Central Africa and confirmed by molecular markers, compared to a neighbour tree with typical leaves of M. excelsa (B). Figure S4. Graphical membership coefficient of Central African individuals of Milicia (genetic clusters K3 to K6) based on nuclear SSR loci. The six colours (blue, black, yellow, pink, green and red) stand for the six genetic clusters. The West African genetic clusters, K1 and K2, were not illustrated but were represented in some individual genome (blue and dark colors). Figure S5. Haplotype network and geographical distribution of trnH-psbA haplotypes in Milicia populations. Figure S6. Haplotype network and geographical distribution of trnC-ycf6 haplotypes in Milicia populations. Figure S7. Ecological niches of the six genetic clusters of Milicia derived from nuclear SSR loci, in the environmental space produced by the principal component analysis method (PCA). The figure above indicates projection of the climatic variables in the plan formed by the two first axes (71.9% of the total variance). Similarly, [file 12862_2016_831_MOESM1_ESM.docx]

**Revealing hidden species diversity in closely related species using nuclear SNPs, SSRs and DNA sequences – A case study in the tree genus *Milicia***

**Additional files**

**Table S1.** Polymorphic (1) and monomorphic (0) SNP loci (P0040, P0095, etc.) in the six genetic clusters of *Milicia* (K1 to K6).

| **Loci** | **K1** | **K2** | **K3** | **K4** | **K5** | **K6** | **Loci** | **K1** | **K2** | **K3** | **K4** | **K5** | **K6** |
| --- | --- | --- | --- | --- | --- | --- | --- | --- | --- | --- | --- | --- | --- |
| P0040 | 1 | 1 | 1 | 1 | 1 | 1 | P0590 | 1 | 1 | 1 | 1 | 1 | 0 |
| P0095 | 1 | 1 | 1 | 1 | 1 | 1 | P0763 | 1 | 0 | 1 | 0 | 1 | 0 |
| P0225 | 1 | 1 | 1 | 1 | 1 | 0 | P0784 | 1 | 1 | 1 | 1 | 1 | 0 |
| P0259 | 1 | 1 | 1 | 1 | 1 | 0 | P0803 | 0 | 1 | 0 | 0 | 0 | 0 |
| P0261 | 0 | 0 | 1 | 1 | 1 | 0 | P0917 | 1 | 1 | 1 | 1 | 0 | 0 |
| P0342 | 1 | 1 | 1 | 1 | 1 | 1 | P0947 | 0 | 0 | 1 | 0 | 1 | 0 |
| P0364 | 1 | 1 | 1 | 1 | 1 | 0 | P1002 | 1 | 0 | 1 | 1 | 1 | 1 |
| P0480 | 1 | 1 | 1 | 1 | 1 | 0 | P1076 | 1 | 1 | 1 | 1 | 1 | 0 |
| P0526 | 1 | 1 | 1 | 1 | 1 | 0 | P1253 | 1 | 1 | 1 | 1 | 1 | 0 |
| P0536 | 0 | 1 | 1 | 1 | 1 | 0 | P1461 | 0 | 1 | 1 | 1 | 1 | 0 |
| P0606 | 1 | 1 | 1 | 1 | 0 | 1 | P1736 | 0 | 1 | 1 | 1 | 1 | 0 |
| P0622 | 0 | 0 | 1 | 1 | 1 | 0 | P1760 | 1 | 1 | 1 | 1 | 1 | 0 |
| P0837 | 1 | 1 | 1 | 1 | 1 | 0 | P2086 | 1 | 1 | 1 | 1 | 0 | 0 |
| P0969 | 1 | 1 | 1 | 1 | 1 | 1 | P2133 | 1 | 1 | 1 | 1 | 1 | 0 |
| P1218 | 1 | 1 | 1 | 1 | 0 | 0 | P2163 | 1 | 1 | 1 | 1 | 0 | 0 |
| P1650 | 1 | 1 | 1 | 1 | 1 | 0 | P2357 | 1 | 1 | 1 | 1 | 1 | 1 |
| P1671 | 1 | 0 | 1 | 1 | 1 | 0 | P2418 | 0 | 0 | 1 | 1 | 1 | 0 |
| P2106 | 0 | 1 | 1 | 1 | 0 | 1 | P2452 | 1 | 0 | 1 | 1 | 1 | 1 |
| P2126 | 1 | 1 | 1 | 1 | 1 | 1 | P2631 | 0 | 1 | 1 | 1 | 1 | 0 |
| P2161 | 1 | 1 | 1 | 1 | 1 | 0 | P2860 | 1 | 1 | 1 | 1 | 0 | 1 |
| P2241 | 1 | 1 | 1 | 1 | 1 | 1 | P2889 | 0 | 1 | 1 | 0 | 0 | 0 |
| P2274 | 1 | 1 | 1 | 0 | 1 | 0 | P2902 | 1 | 1 | 1 | 1 | 1 | 0 |
| P2341 | 1 | 1 | 1 | 1 | 1 | 0 | P2910 | 0 | 1 | 1 | 1 | 1 | 0 |
| P2527 | 1 | 1 | 1 | 1 | 1 | 0 | P3137 | 1 | 1 | 1 | 1 | 0 | 0 |
| P2671 | 1 | 1 | 1 | 1 | 1 | 0 | P3230 | 0 | 1 | 1 | 1 | 1 | 0 |
| P2685 | 1 | 1 | 1 | 1 | 1 | 1 | P3246 | 0 | 1 | 1 | 1 | 1 | 0 |
| P2992 | 1 | 1 | 1 | 1 | 1 | 0 | P3278 | 0 | 0 | 1 | 1 | 1 | 0 |
| P3047 | 0 | 1 | 1 | 1 | 1 | 0 |  |  |  |  |  |  |  |
| P3129 | 1 | 1 | 1 | 1 | 1 | 0 |  |  |  |  |  |  |  |
| P3215 | 0 | 1 | 1 | 1 | 0 | 0 |  |  |  |  |  |  |  |
| P3218 | 1 | 1 | 1 | 1 | 1 | 0 |  |  |  |  |  |  |  |
| P3299 | 1 | 1 | 1 | 0 | 0 | 0 |  |  |  |  |  |  |  |
| P3351 | 1 | 1 | 1 | 1 | 1 | 0 |  |  |  |  |  |  |  |
| P0020 | 1 | 1 | 1 | 1 | 1 | 1 |  |  |  |  |  |  |  |
| P0025 | 1 | 1 | 1 | 1 | 1 | 0 |  |  |  |  |  |  |  |
| P0110 | 1 | 1 | 1 | 1 | 1 | 1 |  |  |  |  |  |  |  |
| P0226 | 0 | 1 | 1 | 1 | 1 | 0 |  |  |  |  |  |  |  |
| P0272 | 1 | 1 | 1 | 1 | 1 | 0 |  |  |  |  |  |  |  |
| P0421 | 1 | 1 | 1 | 1 | 1 | 0 |  |  |  |  |  |  |  |
| P0705 | 0 | 1 | 1 | 1 | 1 | 0 |  |  |  |  |  |  |  |

**Table S2.** Geographic coordinates of Central African individuals of *Milicia* that may represent a new species according to SNP and based on the whole *Milicia* sample of the ITTO Project PD 620/11 Rev.1 (M): “Development and implementation of species identification and timber tracking in Africa with DNA fingerprints and stable isotopes” (1,833 individuals). Individuals in bold are those selected for the SNP-SSR comparison (435 individuals in total) and which all turned out to be representatives of a new species as the nuclear gene *At103* confirmed.

| **Country** | **Sample name** | **Latitude** | **Longitude** | **Country** | **Sample name** | **Latitude** | **Longitude** |
| --- | --- | --- | --- | --- | --- | --- | --- |
| Cameroon | C_01_11 | 2.4465 | 10.4744 | DR of Congo | DRC_14_03 | 0.1596 | 20.4524 |
| Congo Brazzaville | CB_01_01 | 3.2763 | 16.7489 | DR of Congo | DRC_14_04 | 0.1609 | 20.4499 |
| Congo Brazzaville | CB_01_02 | 3.1383 | 17.0119 | DR of Congo | DRC_14_06 | 0.1594 | 20.4440 |
| **Congo Brazzaville** | **CB_01_02B** | **2.8398** | **17.0647** | **DR of Congo** | **DRC_15_07** | **1.4976** | **21.0478** |
| Congo Brazzaville | CB_01_04 | 3.0097 | 17.0278 | DR of Congo | DRC_19_01 | -1.9041 | 28.4561 |
| Congo Brazzaville | CB_01_04B | 2.9188 | 17.0271 | **DR of Congo** | **DRC_19_02** | **-1.9039** | **28.4543** |
| Congo Brazzaville | CB_01_05B | 2.9439 | 17.0274 | **DR of Congo** | **DRC_19_03** | **-1.8674** | **28.4511** |
| Congo Brazzaville | CB_01_06 | 3.0891 | 16.9697 | **DR of Congo** | **DRC_19_04** | **-1.8964** | **28.4553** |
| Congo Brazzaville | CB_01_06B | 3.0651 | 16.9976 | DR of Congo | DRC_19_05 | -1.8897 | 28.4537 |
| Congo Brazzaville | CB_01_07 | 3.0868 | 16.9752 | DR of Congo | DRC_19_06 | -1.8913 | 28.4543 |
| Congo Brazzaville | CB_01_07B | 3.0938 | 16.9575 | DR of Congo | DRC_19_07 | -1.8688 | 28.4510 |
| Congo Brazzaville | CB_01_08 | 3.1001 | 16.9404 | **DR of Congo** | **DRC_19_08** | **-1.8674** | **28.4511** |
| Congo Brazzaville | CB_01_08B | 3.2476 | 16.7679 | **DR of Congo** | **DRC_19_09** | **-1.8664** | **28.4477** |
| Congo Brazzaville | CB_01_09 | 3.0959 | 16.9519 | DR of Congo | DRC_19_10 | -1.8730 | 28.4519 |
| Congo Brazzaville | CB_01_09B | 3.1394 | 16.9673 | DR of Congo | DRC_19_11 | -1.8665 | 28.4476 |
| Congo Brazzaville | CB_01_11 | 3.1353 | 17.0026 | DR of Congo | DRC_19_12 | -1.8639 | 28.4499 |
| Congo Brazzaville | CB_01_12 | 3.2845 | 16.8022 | DR of Congo | DRC_19_13 | -1.8627 | 28.4501 |
| Congo Brazzaville | CB_01_13 | 3.0908 | 16.9657 | DR of Congo | DRC_19_14 | -1.8639 | 28.4496 |
| Congo Brazzaville | CB_18_01 | -2.9078 | 12.6455 | DR of Congo | DRC_19_15 | -1.8737 | 28.4521 |
| Congo Brazzaville | CB_18_02 | -3.1732 | 13.8152 | DR of Congo | DRC_19_16 | -1.8898 | 28.4531 |
| Congo Brazzaville | CB_18_03 | -3.1593 | 13.8164 | DR of Congo | DRC_19_17 | -1.8915 | 28.4549 |
| Congo Brazzaville | CB_18_04 | -3.1582 | 13.8143 | DR of Congo | DRC_19_18 | -1.8930 | 28.4553 |
| **Congo Brazzaville** | **CB_18_06** | **-3.1596** | **13.8171** | DR of Congo | DRC_19_19 | -1.8930 | 28.4553 |
| **Congo Brazzaville** | **CB_18_07** | **-3.1645** | **13.8216** | DR of Congo | DRC_19_20 | -1.8891 | 28.4530 |
| **Congo Brazzaville** | **CB_18_08** | **-3.1756** | **13.8253** | **DR of Congo** | **DRC_20_03** | **-3.0617** | **28.1823** |
| **Congo Brazzaville** | **CB_18_09** | **-3.1832** | **13.8240** | DR of Congo | DRC_27_18 | -0.1807 | 25.6247 |
| Congo Brazzaville | CB_18_10 | -3.1882 | 13.8199 | DR of Congo | DRC_27_19 | -0.1699 | 25.6087 |
| Congo Brazzaville | CB_18_12 | -3.1761 | 13.8149 | DR of Congo | DRC_28_16 | -0.8128 | 26.4791 |
| Congo Brazzaville | CB_18_13 | -3.1739 | 13.8147 | Gabon | G_13_03 | -0.0118 | 13.6619 |
| Congo Brazzaville | CB_18_14 | -3.1713 | 13.8143 | Gabon | G_13_10 | -0.0098 | 13.6608 |
| Congo Brazzaville | CB_18_19 | -3.1810 | 13.8270 | Gabon | G_13_14 | -0.0114 | 13.6609 |
| Congo Brazzaville | CB_18_20 | -3.1784 | 13.8232 |  |  |  |  |


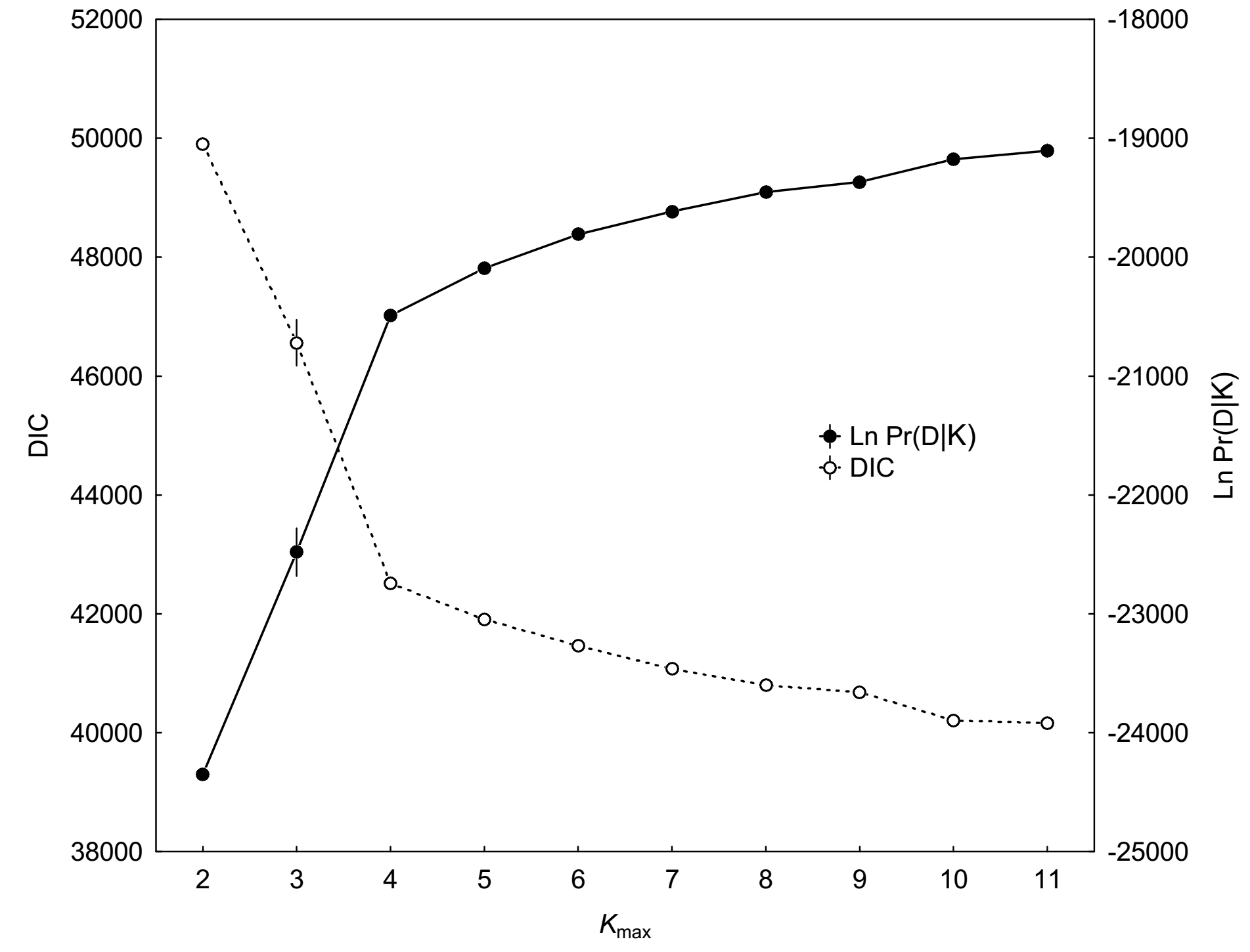


**Figure S1.** Determination of the number of genetic clusters in *Milicia* populations based on SNPs genotypes.


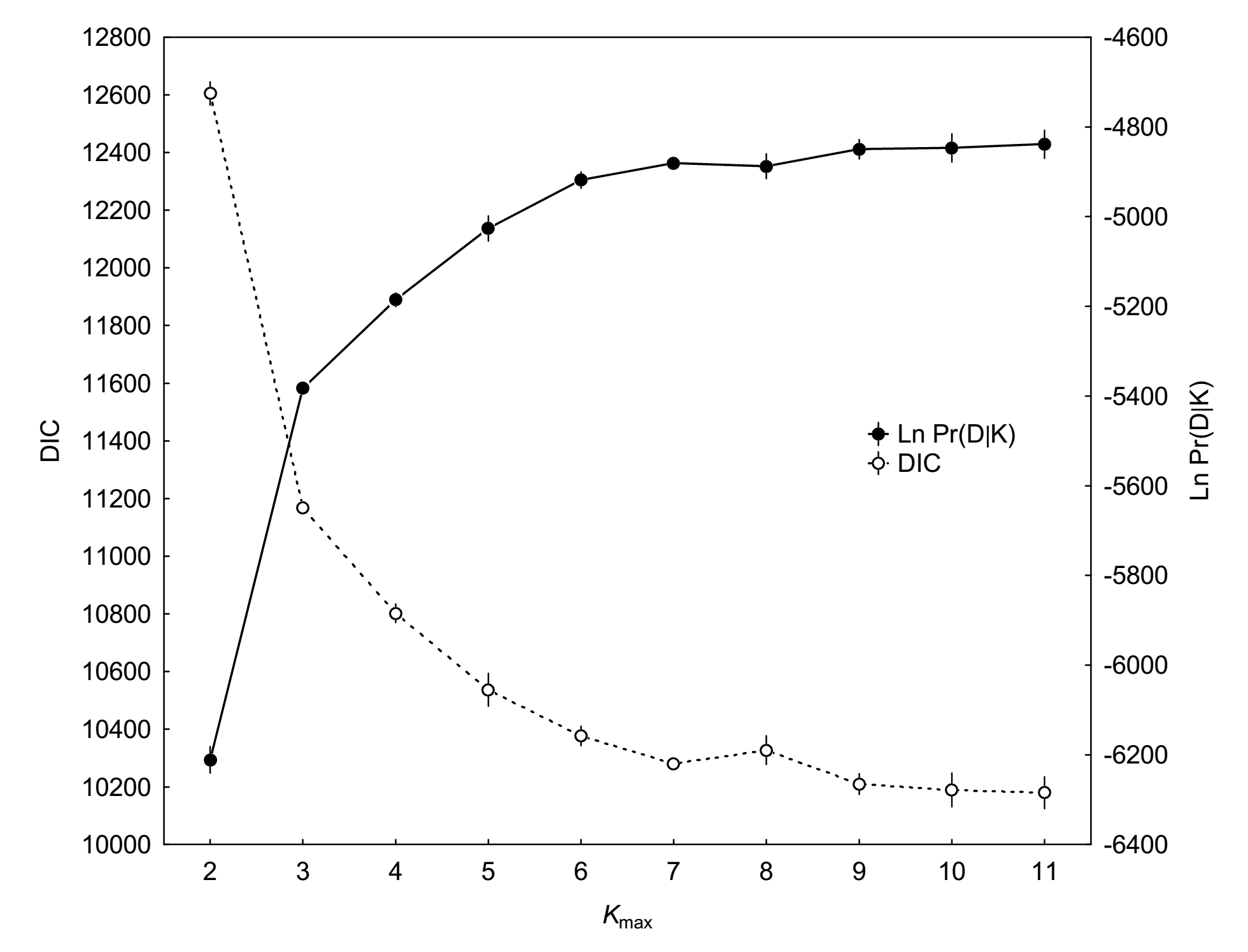


**Figure S2**. Determination of the number of clusters in Milicia populations based on SSRs genotypes

| (A)  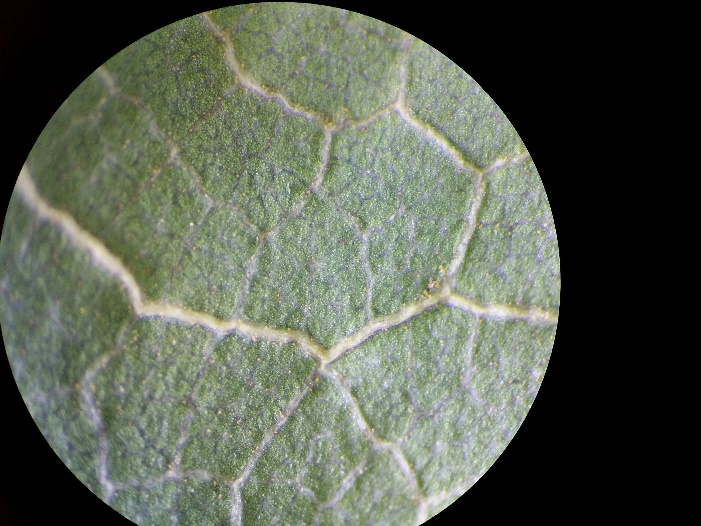 | (B)  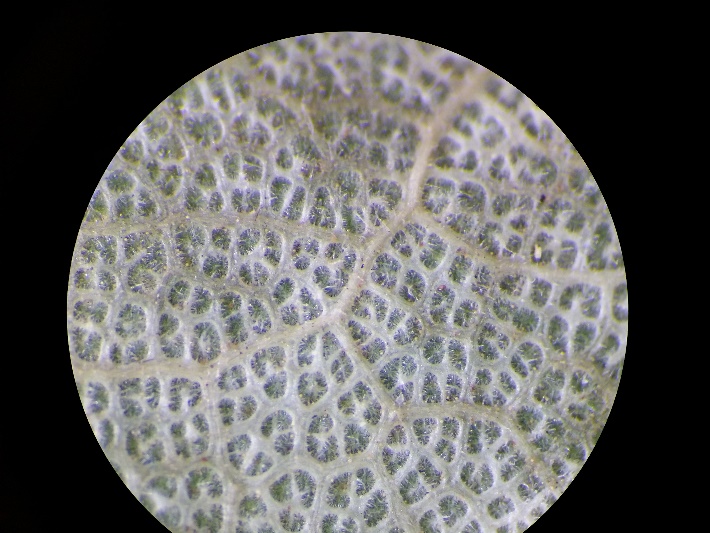 |
| --- | --- |

**Figure S3**. (A) Lower surface of leaf for a tree morphologically identified as *M. regia* in Central Africa and confirmed by molecular markers, compared to a neighbour tree with typical leaves of *M. excelsa* (B).


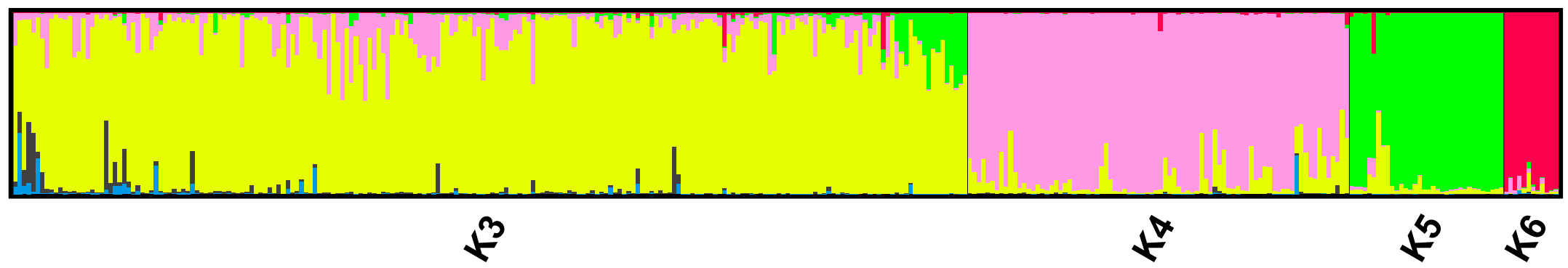


**Figure S4**. Graphical membership coefficient of Central African individuals of *Milicia* (genetic clusters K3 to K6) based on nuclear SSR loci. The six colours (blue, black, yellow, pink, green and red) stand for the six genetic clusters. The West African genetic clusters, K1 and K2, were not illustrated but were represented in some individual genome (blue and dark colors).


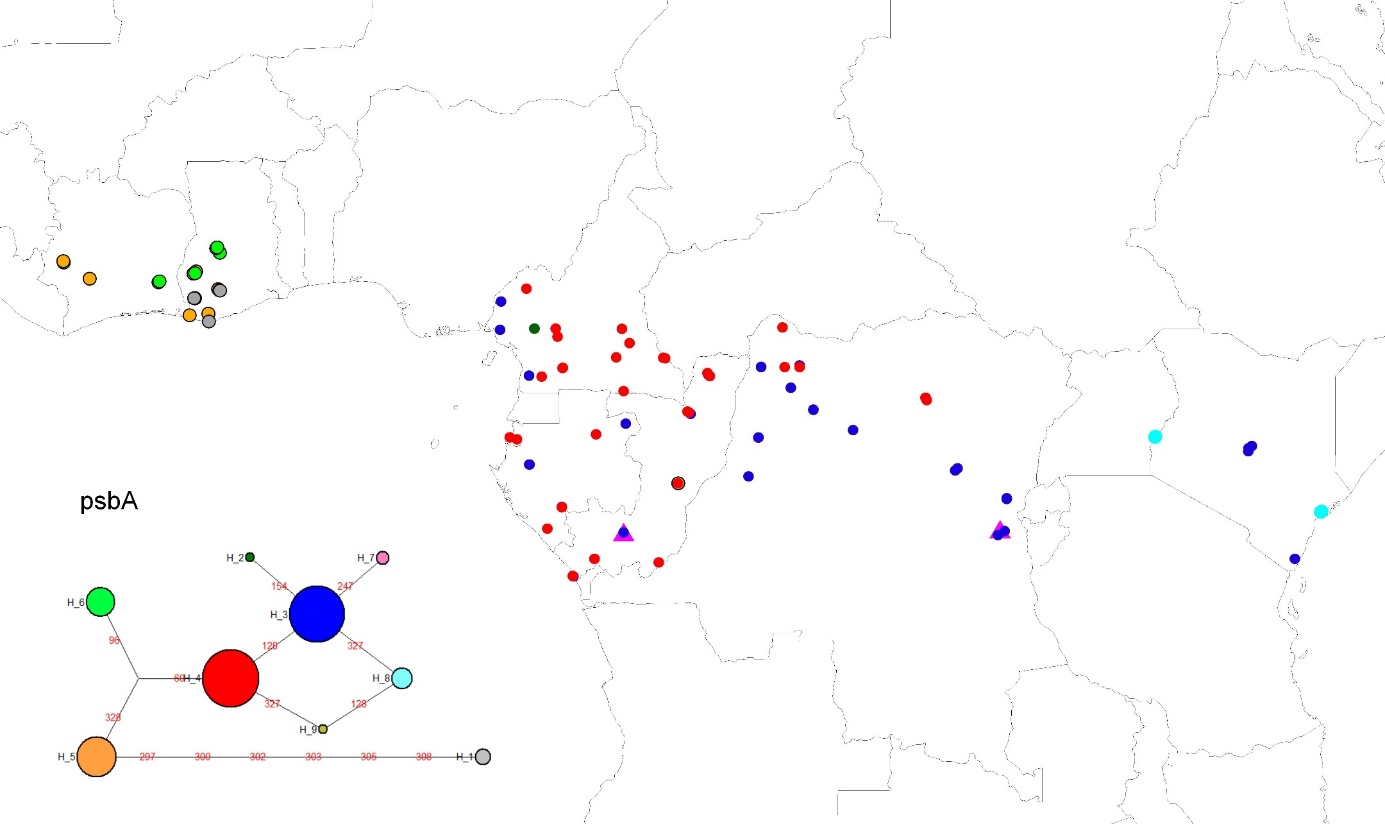


**Figure S5**. Haplotype network and geographical distribution of *trnH-psbA* haplotypes in *Milicia* populations


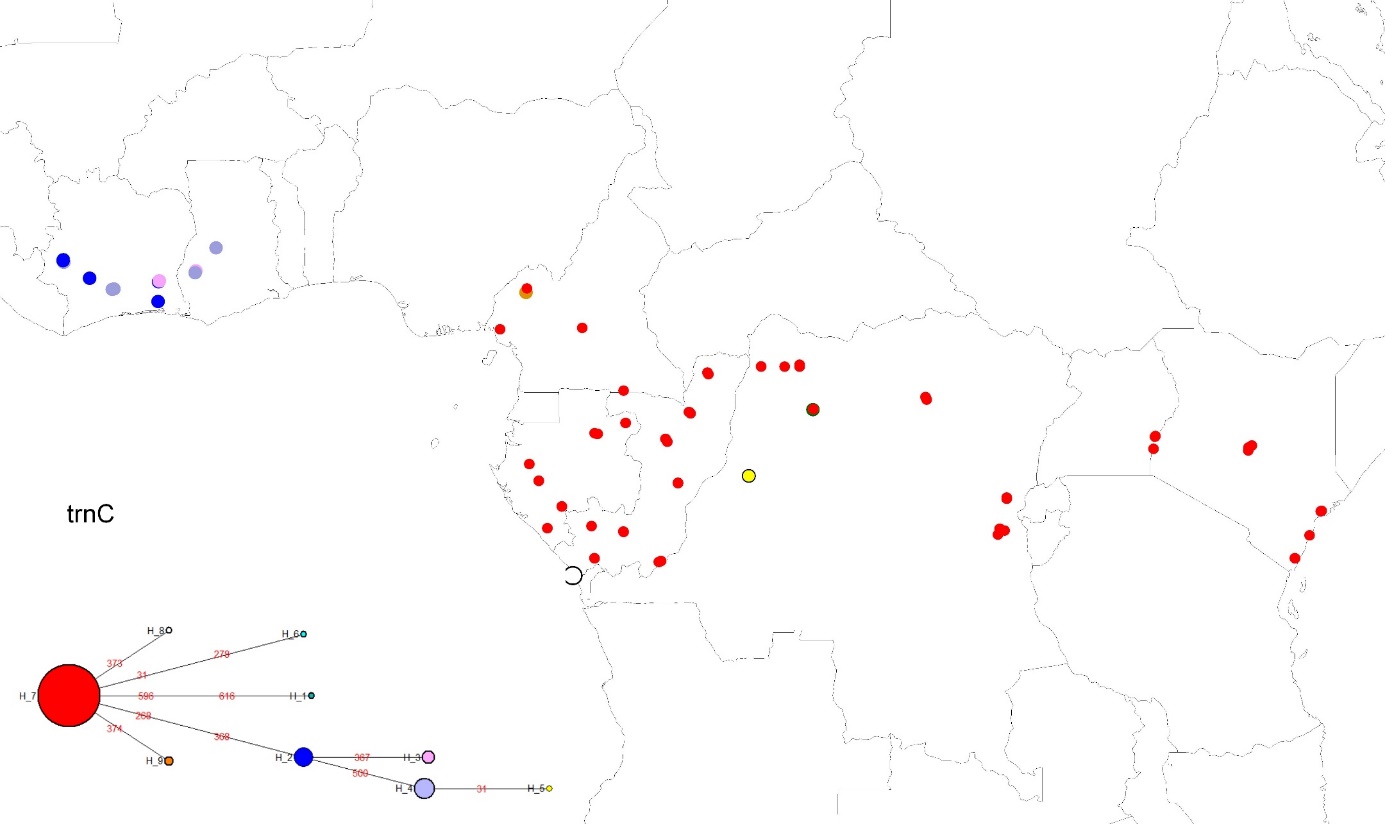


**Figure S6**. Haplotype network and geographical distribution of *trnC-ycf6* haplotypes in *Milicia* populations

.


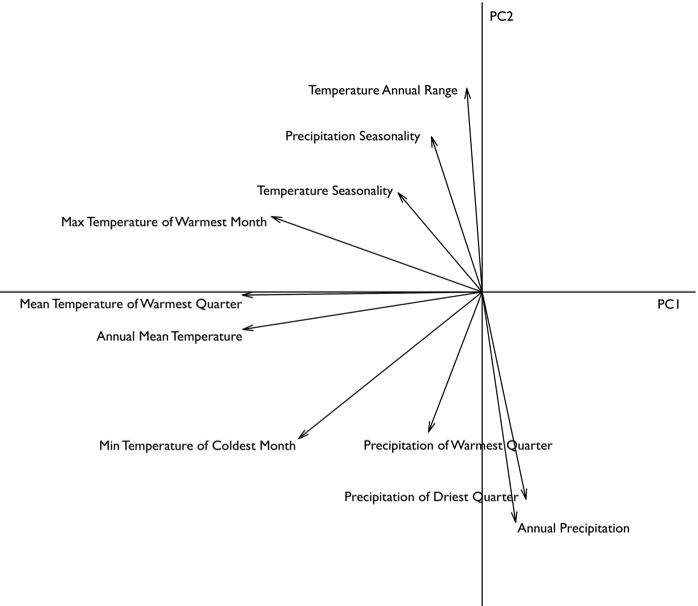


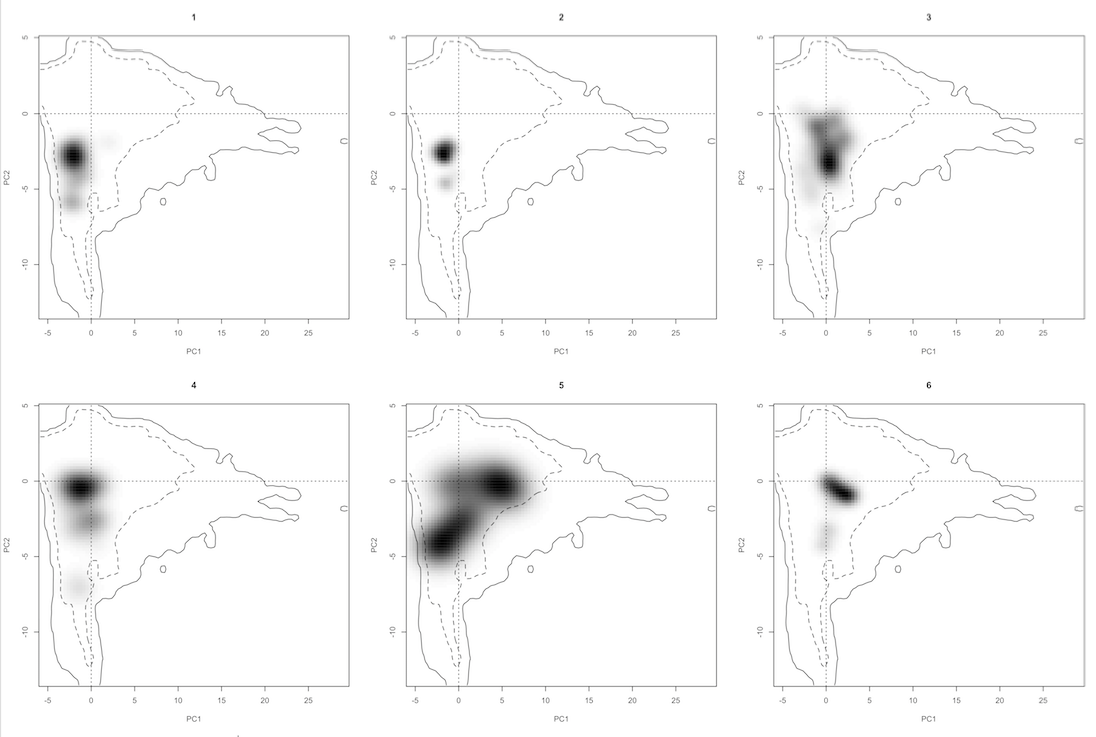


**Figure S7**. Ecological niches of the six genetic clusters of *Milicia* derived from nuclear SSR loci, in the environmental space produced by the principal component analysis method (PCA). The figure above indicates projection of the climatic variables in the plan formed by the two first axes (71.9% of the total variance). Similarly, the grey-to-black shading in the six small figures (1 = cluster K1; 2 = cluster K2; etc.) represents the grid cell density (black being the highest density) of the concerned genetic cluster in the PCA plan. The first dashed curve represents the 50% of the available environment space whereas the solid line stands for the entirety of the species environment.

| K1  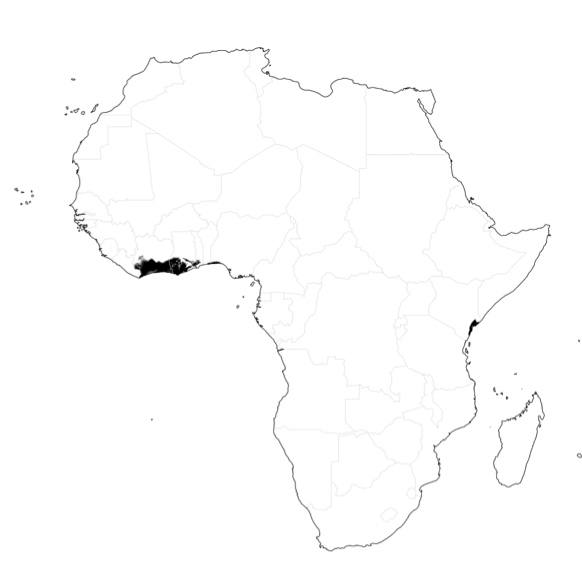 | K2  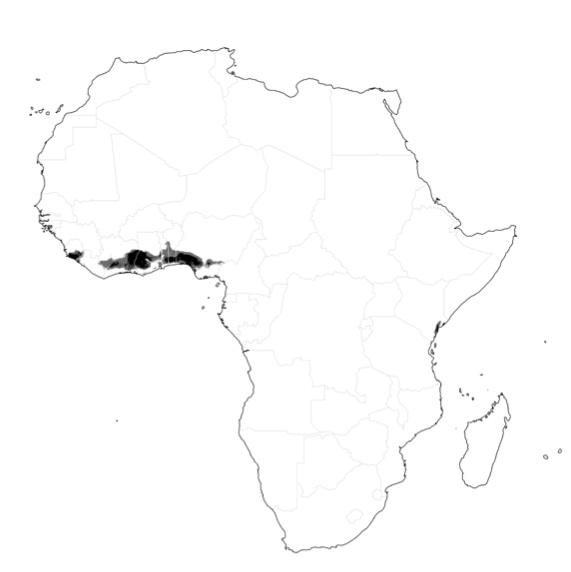 | K3  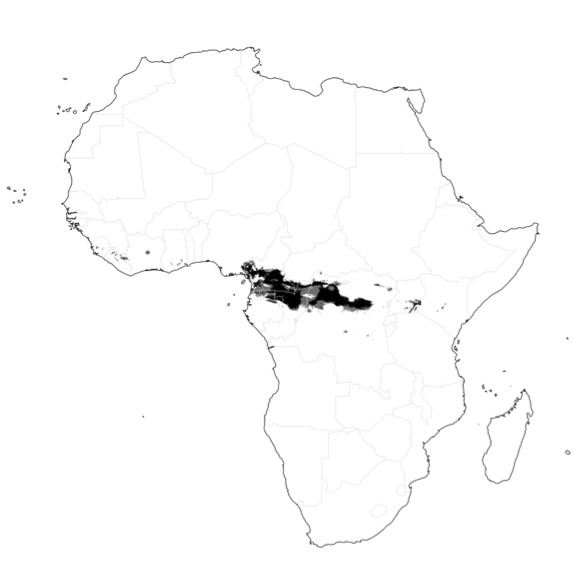 |
| --- | --- | --- |
| K4  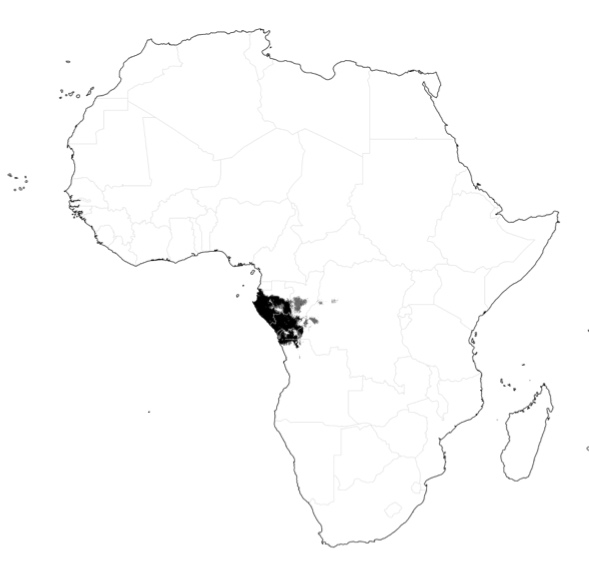 | K5  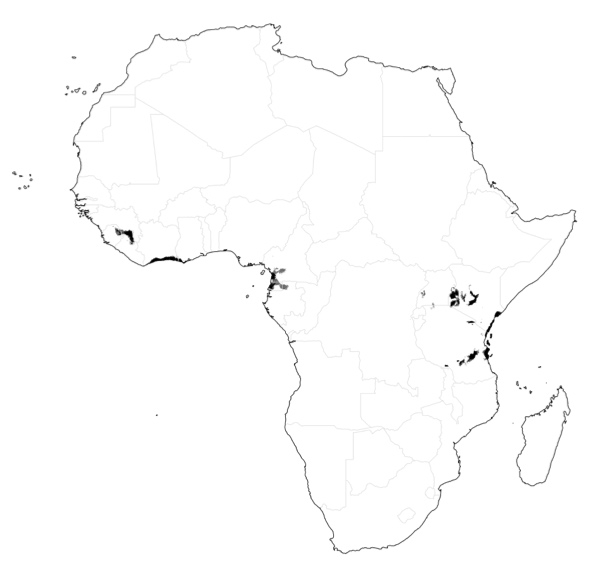 | K6  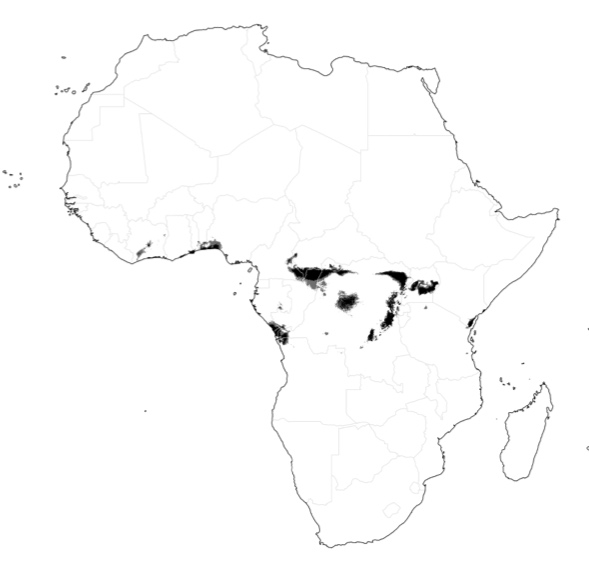 |

**Figure S8**. Potential distribution range of *Milicia* genetic clusters during the Last Glacial Maximum (LGM) according to niche modelling through Maxent approach. Black areas represent predicted regions with a probability higher than 50%.
